# Points in Regard to Phthisis Pulmonalis

**Published:** 1907-05-18

**Authors:** 


### SPECIAL ARTICLE.

### POINTS IN REGARD TO PHTHISIS PULMONALIS.

### I. The Proportion of Cases Without Expectoration.

There is always room for doubt whether abnormal physical signs, present at the apex of a lung, are due to phthisis until the existence of a tuberculous lesion is absolutely proved by the occurrence of tubercle bacilli in the sputum. It occasionally happens that physical signs and symptoms which at first sight appear typical of phthisis, ultimately turn out to be due to something quite different. To give a single example. A patient may present himself complaining of recurrent hæmoptysis; upon examination, impairment of note may be found over the apex of the upper lobe of the right lung, together with crackling rales, bronchophony, and prolongation of the expiratory part of the vesicular murmur; the commonest cause of hæmoptysis and of these physical signs is no doubt phthisis, but occasionally a small thoracic aneurysm may produce exactly the same results by partially obstructing the bronchus going to the upper lobe of the right lung. The diagnosis of phthisis should never be made without confirming it, where possible, by an examination of the sputum for tubercle bacilli.

It at once becomes important to know what proportion of cases of phthisis do not expectorate anything from the lung. Statistics upon this point are bound to vary according to the kind of patients who come under treatment, the length of time they are kept under observation, and so on; but it is remarkable how many patients who have made a comparatively long stay at a sanatorium have had no expectoration throughout their stay there. The following figures are obtained from the annual report for the Mount Vernon Hospital for Consumption at Hampstead; the extent of the lung trouble was gauged by the physical signs, and it was found that of 198 patients who had evidence of infiltration of one lobe only, 35 had no expectoration; of 277 cases with infiltration of more than one lobe, but without any physical signs to indicate cavitation, 25 had no expectoration; and of 203 in whom there were cavities in the lung, 10 had no expectoration. Expressed as percentages we find that of cases with infiltration of one lobe only, 18 per cent. had no expectoration; of cases with infiltration of more than one lobe, but without cavitation, 9 per cent. had no expectoration; of cases with cavitation, 5 per cent. had no expectoration.

It naturally follows that in all such instances one has to be content with diagnosing phthisis from the symptoms and physical signs alone, without obtaining positive proof of the lesion being tuberculous.

Presumably there was sputum really coming from the lung in at least some of these patients; but it was swallowed instead of being expectorated, and the patient could not change the swallowing into expectoration when desired. Acting upon this assumption, it was thought that traces of sputum might sometimes be found adhering to the larynx, and that such fragments might be recovered by the use of a laryngeal probe. This was tested in a few cases, and although the attempts so far have not been very successful, the investigation seems worthy of a more extended trial. The difficulties are, mainly, that the amount of sputum to be obtained from the larynx is very small, and that it is difficult to remove the probe without contaminating it with the secretions in the pharynx. That the method is feasible was shown by the positive results obtained in cases already known to be positive; but Dr. Roome did not succeed in getting a positive result from any patient who never expectorated.

### II. The Proportion of Cases of Phthisis in Which Tubercle Bacilli are not Found in the Sputum.

There are many reasons why tubercle bacilli may not be present in the sputum of a phthisical patient. We need not enter into all of these here, but we may mention two of the commoner causes. The first depends upon the fact that there is usually some reactionary bronchitis in the non-tuberculous lung tissue immediately round the tuberculous foci; muco-pus coughed up may be the local bronchitic secretion, and therefore free from any actual tuberculous material. It usually happens, of course, that both tuberculous and non-tuberculous muco-pus are expectorated together, but it is quite possible for the simple bronchitic sputum to preponderate so much that, without very special methods of examining the expectoration, no tubercle bacilli are found. It is well known that the sputum in cases of acute miliary tuberculosis of the lungs seldom contains tubercle bacilli, and very possibly the explanation is that just suggested. The second reason is that in many cases of phthisis, particularly those of old date, there is a secondary infection of the bronchioles and cavities by all sorts of other organisms, staphylococci, streptococci, diplococci, and other bacteria of various kinds. Many of the symptoms of phthisical cachexia are due to the toxic effects of these other organisms, and not to the tubercle bacilli at all. The tubercle bacilli have damaged certain areas of the lung and the other organisms infect these areas afterwards, and produce a chronic suppuration with its attendant consequences. The sputum may be abundant, but tubercle bacilli may be difficult to find, and not infrequently they may not be found at all. The Mount Vernon Hospital statistics show that out of 163 patients who had infiltration of one lobe only, and who expectorated, 93 had no tubercle bacilli discovered in their sputum notwithstanding repeated examinations; of 152 patients who had infiltration of more than one lobe without signs of cavitation, and who expectorated, 54 proved negative as regards tubercle bacilli in their sputum; and not even every patient who had extensive phthisis with obvious cavitation showed tubercle bacilli in the sputum, 11 out of 193 being negative. Expressed as percentages we find that of patients with infiltration of one lobe only and who expectorated, 57 per cent. had no discoverable tubercle bacilli in the sputum; of cases with infiltration of more than one lobe, without obvious cavitation, 22 per cent. had no discoverable tubercle bacilli in the sputum; of cases with cavitation, 5 per cent. had no discoverable tubercle bacilli in the sputum.

Taking the cases all round, independently of the extent of the lesion, the total number of cases was 678, and out of every 100 there were 10 who had no expectoration at all, 24 who had expectoration in which no tubercle bacilli could be found, while in 66 only could tubercle bacilli be detected in the sputum. In other words, all important though it is that tubercle bacilli should be found if a diagnosis of phthisis is to be absolutely certain, we have to be content to know that, even in a special hospital, about one-third of all the cases have to be diagnosed without these bacilli being found.

### III. The Association of Heart Disease with Phthisis.

It is usually taught that heart disease, other than congenital malformation, and phthisis seldom occur in one and the same patient. This is undoubtedly true in the main. It is probably not because there is any inherent protective influence exerted by such a lesion as mitral stenosis upon the lung, or by phthisis pulmonalis on the heart. It is much more likely that the rheumatic diathesis and the tuberculous diathesis seldom occur simultaneously in the same patient. There can be little doubt that some persons are born predisposed to phthisis; there is almost as little doubt that other persons are born with a special tendency to suffer from acute rheumatism. It is very difficult to give any explanation of these hereditary tendencies or "diatheses," but that they are of great significance we cannot doubt. It is not that the person with the rheumatic diatheses *cannot* develop phthisis, it is merly that he is not very likely to do so. It is not surprising, therefore, to find that heart disease and phthisis pulmonalis do sometimes occur in the same patient. The extent of this liability is shown by a series of 727 consecutive consumptive patients, of whom 20, or rather less than 3 per cent., had heart disease as well.

### IV. The Other Tuberculous Lesions which Phthisical Patients may Suffer From.

It is distinctly rare for phthisical patients to suffer from tubercle elsewhere than in the *lung* and *pleura*, except in those parts to which the tuberculous sputum may be directly carried, namely the *larynx*, the *intestine*, and much more rarely the *buccal cavity*. The patient may develop miliary tuberculosis of the lung; but for him to develop general miliary tuberculosis all over the body is a pathological rarity. Tuberculous meningitis, for example, is very rare in connection with phthisis pulmonalis.

Dr. Nathan Raw goes so far as to say that there are two forms of tuberculosis in man, the *human* type and the *bovine* type. The human type, due to bacilli from another human being, leads, he says, to tuberculous lung, pleura, larynx, and bowel; whereas he holds that other tuberculous lesions, such as lupus, caseous lymphatic glands, arthritis, spinal caries, and so on, up to general tuberculosis, are due to bacilli that have come from a bovine origin. This may very possibly be true in the main; but in a certain number of cases of phthisis tuberculous lesions occur elsewhere than in the lungs, pleura, larynx and intestine. Thus, out of a series of 727 consecutive cases of phthisis in the Mount Vernon Hospital, there were:—

Four cases with spinal caries.
Two cases with tuberculous kidney.
Two cases with tuberculous ulceration of the bladder.
One case with lupus.
One case with tuberculous dactylitis.
One case with tuberculous arthritis.
One case with tuberculous abscess in the arm; and
One case with acute miliary tuberculosis.

These would doubtless be explained by Dr. Nathan Raw as cases of phthisis in which abundant milk diet had led to an infection with bovine tubercle in addition to the existing human infection. Very possibly this is so. In any case, the proportion of cases with what we may call extra-lesions is small, and really helps to support Dr. Nathan Raw's contention. The only tuberculous lesions that are at all common in consumptive patients, besides those in the lungs, are tuberculous laryngitis and tuberculous ulceration of the bowel. Symptoms of the latter are usually conspicuous by their absence, and it may be worth noting, in conclusion, that it is almost unknown in the post-mortem room not to find tuberculous ulcers in the bowel in all patients who have had tuberculous laryngitis during life.

